# Supplementary material for: A biallelic SNIP1 Amish founder variant causes a recognizable neurodevelopmental disorder
Source: PLoS Genet. 2021 Sep 27;17(9):e1009803. doi: 10.1371/journal.pgen.1009803 (PMC8496849; doi:10.1371/journal.pgen.1009803)
Supplement: S1 Text — (DOCX) [file pgen.1009803.s001.docx]

**Patient EEG report findings for eight individuals affected with *SNIP1*-related disorder**

**Individual 1**

One hour EEG at 11 months, parental concern about possible seizures:

*“Abnormal awake and asleep EEG due to a left posterior sharp and slow wave focus seen in sleep.”*

20 hour EEG at 17 months, known epilepsy, unmedicated, admitted for status epilepticus and respiratory failure:

*“…focal slowing in left hemisphere with focal epileptiform discharges on T5/01, asymmetric sleep structures overlying continuous slow activity. No seizures were seen. These findings are consistent with a moderate encephalopathy, focal dysfunction or structural lesion in the left hemisphere and a potentially epileptogenic focus in the left temporo-occipital region.”*

**Individual 2**

44 hour EEG day 1 and 2 of life:

*“excessive background discontinuity, asynchrony and frequent multifocal discharges, most common in the left centroparietal region as well as recurrent, prolonged focal seizures from same region.”*

**Individual 3**

Prolonged EEG at 16 months:

*“global cerebral dysfunction, right greater than left with active biposterior epileptiform activity consistent with seizure focus. These findings are concerning for genetic epileptic encephalopathy.”*

**Individual 4**

EEG as part of sleep study:

*“slow in general, sleep wake stages were difficult to distinguish.”*

**Individual 5**

EEG at 11 months:

*“abnormal awake and asleep EEG due to presence of electroclinical seizure. Seizures originated in left and right temporal region.”*

**Individual 6**

EEG at 4 years:

*“frequent multifocal discharges, numerous subclinical seizures and a few tonic seizures. Multiple areas of cortical irritability consistent with active epilepsy.”*

**Individual 7**

EEG at 3 months:

***“****Normal”*

At 10 months:

*“prominent right sided slowing, diminished voltage of beta activity on the right, diminished sleep spindle, generally slower waking background. No epileptiform features”*

2 years old:

***“****abnormal awake and asleep EEG given lack of well defined posterior dominant rhythm, absence of sleep structures, left hemispheric slowing with embedded sharp waves, no seizures are seen. Findings suggest a structural or functional epileptogenic lesion in the left hemisphere.”*

**Individual 8**

EEG at 15 months:

*“This is an abnormal EEG due to the diffuse background slowing as well as the decrease amplitude on the left. This suggests diffuse cerebral dysfunction which may be more severe on the left side. There is no epileptiform activity present in this record.”*

At 18 months old:

*“EEG was abnormal due to excessive fast activity. There were no epileptiform discharges
present in this record.”*

At 21 months old:

*“The EEG was abnormal due to left temporal sharp waves, generalized background slowing,
worse on the left and asymmetry of the background, specifically decreased fast activity on the left.”*
