# Supplementary material for: A biallelic SNIP1 Amish founder variant causes a recognizable neurodevelopmental disorder
Source: PLoS Genet. 2021 Sep 27;17(9):e1009803. doi: 10.1371/journal.pgen.1009803 (PMC8496849; doi:10.1371/journal.pgen.1009803)
Supplement: S2 Text — (DOCX) [file pgen.1009803.s002.docx]

**ACMG Variant Classification Criteria**

| Gene: | ***SNIP1*** |
| --- | --- |
| Variant description: HGVS | **NM_024700.4:c.1097A>G, p.(Glu366Gly)** |
| Variant location: GRCh38 (hg38): | **Chr1(GRCh38):g.37537842T>C** |
| Variant classification: | **Pathogenic** |

| **Evidence for variant classification using ACMG/AMP guidelines**  (Evidence code_ level) ([Richards *et al* 2015 Genet Med](https://www.ncbi.nlm.nih.gov/pubmed/25741868))[[1](#_ENREF_1)] |
| --- |

This variant is currently listed in Clinvar as a Variant of Uncertain Significance (Accession ID:VCV000030717.3, accessed on August 26^th^ 2021) as there has previously been felt to be insufficient evidence to classify the variant as pathogenic.

- Two independent series of functional evaluation of the outcome on the protein at the level of the variant have been undertaken (**PS3_Strong**):
  - *In vitro* functional studies of the variant in transfected mIMCD3 cells resulted in instability of the protein product on Western blotting and differences in localisation with a more aggregated appearance (Puffenberger EG, et al. 2012 PLoS One. 7(1):e28936)[[2](#_ENREF_2)].
  - The current study provides transcriptome studies from individuals homozygous for the variant that show significantly differential expression of several molecular signalling pathways. We believe that the combination of the results of these functional studies demonstrate this variant has pathogenic effects on normal cellular processes and is sufficient to warrant the use of classification evidence level.
- This variant has been reported at a low frequency in the gnomAD database v3.1 (11 heterozygotes (10 Amish) in 152,214 individuals (allele frequency 0.00007227), no homozygotes) (**PM2_Moderate**).
- The phenotype of *SNIP1*-related disorder is distinctive and highly recognisable. The variant segregates as appropriate for an autosomal recessive disorder amongst 35 affected Amish individuals, their parents and unaffected siblings. Due this compelling segregation data we have used a higher evidence level (Jarvik and Browning, 2016)[[3](#_ENREF_3)] (**PP1_Strong**).
- The variant is highly evolutionarily conserved (Figure 1G) and multiple lines of computational evidence support a deleterious effect on the gene or gene product

[SIFT (0.001), Provean (-6.76) and Polyphen2 (humvar 0.966) predict the variant as deleterious] (**PP3_Supporting**).

**References**

1. Richards, S., et al., *Standards and guidelines for the interpretation of sequence variants: a joint consensus recommendation of the American College of Medical Genetics and Genomics and the Association for Molecular Pathology.* Genet Med, 2015. **17**(5): p. 405-24.

2. Puffenberger, E.G., et al., *Genetic mapping and exome sequencing identify variants associated with five novel diseases.* PLoS One, 2012. **7**(1): p. e28936.

3. Jarvik, G.P. and B.L. Browning, *Consideration of Cosegregation in the Pathogenicity Classification of Genomic Variants.* Am J Hum Genet, 2016. **98**(6): p. 1077-1081.
