# Supplementary material for: A biallelic SNIP1 Amish founder variant causes a recognizable neurodevelopmental disorder
Source: PLoS Genet. 2021 Sep 27;17(9):e1009803. doi: 10.1371/journal.pgen.1009803 (PMC8496849; doi:10.1371/journal.pgen.1009803)
Supplement: S1 Fig — (DOCX) [file pgen.1009803.s003.docx]

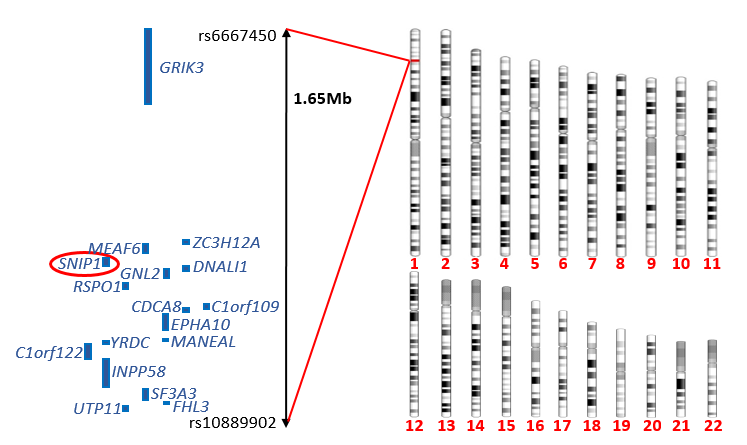


**S1 Fig:** **Genome-wide SNP mapping in four affected individuals** (X:30, X:31, XI:6 and XI:7) identified a single (1.65Mb) region of shared homozygosity, containing 17 genes.
