# Supplementary material for: A biallelic SNIP1 Amish founder variant causes a recognizable neurodevelopmental disorder
Source: PLoS Genet. 2021 Sep 27;17(9):e1009803. doi: 10.1371/journal.pgen.1009803 (PMC8496849; doi:10.1371/journal.pgen.1009803)
Supplement: S3 Fig — Map created in GTex Portal, Broad Institute 2021. (DOCX) [file pgen.1009803.s005.docx]

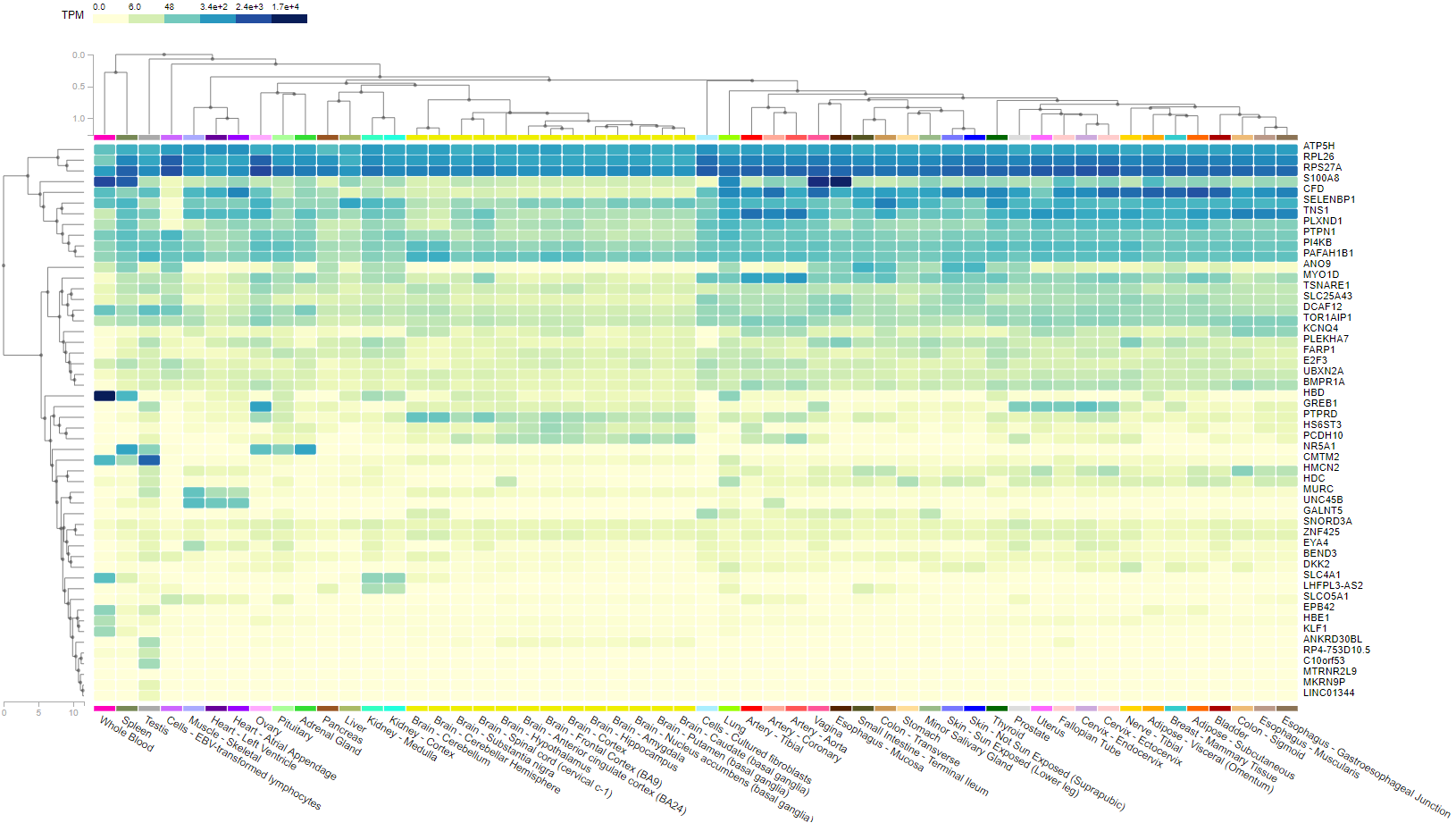
**S3 Fig:** **Heatmap showing tissue expression levels of the top 50 genes with downregulated expression in individuals with *SNIP1*-related disorder.** Map created in GTex Portal, Broad Institute 2021.
