# Supplementary material for: A biallelic SNIP1 Amish founder variant causes a recognizable neurodevelopmental disorder
Source: PLoS Genet. 2021 Sep 27;17(9):e1009803. doi: 10.1371/journal.pgen.1009803 (PMC8496849; doi:10.1371/journal.pgen.1009803)
Supplement: Attachment — Submitted filename: Response to reviewers_SNIP1_PLOS Genetics.docx [file pgen.1009803.s009.docx]

Professor Gregory S. Barsh and Professor Gregory P. Copenhaver

Editors-in-chief,

*PLOS Genetics*

*25^th^ August 2021*

Dear Professors Barsh and Copenhaver,

Re: Manuscript entitled **‘A biallelic *SNIP1* Amish founder variant causes a recognizable neurodevelopmental disorder’**, Ammous *et al.*

We were delighted to receive the reviewers’ positive remarks on our manuscript and have addressed their questions and comments below.

**Reviewer 1:**

*Ammous, et al.* describe a large cohort of Amish individuals with homozygous variation affecting one amino acid residue of SNIP1. While two publications have previously associated this gene with a complex NDD (with recognizable clinical features including seizures, craniofacial features, etc.), this paper adds detailed phenotypic data for many additional individuals. The authors also performed RNA-seq on a subset of individuals and suggest pathways involved in pathogenesis. The strengths of the manuscript include detailed phenotypic data for a large cohort, allowing creation of care recommendations. The study also provides a notable (although small) RNA-seq dataset that may aid in additional research opportunities. My concerns are mostly regarding the novelty of the gene-disease relationship; I would suggest changing the title of the paper to reflect previous work that has been done in this area, and that this is an expansion of that work. Also, it is not clear if the 3 individuals from Puffenberger, et al. were included as part of the 35 here, although it seems like at least one is. Fig1E here looks identical to that of Puffenberger, et al.’s Figure 3B. De-identified images that were covered in the pdf may also be replicated. It would be nice to show data in that Figure from probands other than those that have been previously published, and generally distinguish what was previously done from what is new here.

We have altered the title of the manuscript to **‘A biallelic *SNIP1* Amish founder variant causes a recognizable neurodevelopmental disorder’**, to clarify that our manuscript is confirming *SNIP1* as a disease gene and delineating the full phenotype of ‘*SNIP1*-related disorder’. In addition to the references we originally made to the Puffenberger et al (2012) paper in our manuscript, we have altered the abstract and discussion to clearly state that we are confirming the disease-gene/variant relationship previously postulated in that manuscript. We have also now made it clear that a comprehensive clinical evaluation for two of the affected individuals described in that paper are also included within our study (results section). We have altered Figure 1 to include images of four individuals who have not been previously published clearly illustrating the recognizable facial phenotype of *SNIP1*-related disorder, and we have removed Figure 1E which was duplicated in our original submission (we apologise for that oversight).

1. Either add the RefSeq NM and version, or remove the c./p. when just referencing the variant throughout the text. Do leave all the details on p.11, line 196.

We have now amended our reference to the variant within the text to either include the RefSeq identifier NM_024700.4:c.1097A>G p.(Glu366Gly), or have referred to just the ‘variant’ removing the c. and p. nomenclature.

2. change “consolidate” in abstract (further support? Solidify?)

We have changed the word ‘consolidate’ and expanded the sentence to ‘confirm the *SNIP1* gene variant’ to reflect this point.

3. Overall I would like clarification on the number of individuals and their relatedness. I was a bit confused by the sample numbers at the beginning of the discussion. Why weren’t all 49 individuals who had homozygous SNIP1 variation included? Due to limitations of clinical info? It may be useful to move this summary to the beginning of the results to clarify the population.

We identified 51 individuals affected with suspected *SNIP1*-related disorder, of whom 49 were genotyped. The two individuals not genotyped both displayed characteristic clinical features of the disorder and had an affected sibling confirmed to be homozygous for the *SNIP1* founder variant, and so are thus also very likely to have the condition and their parents opted not to confirm this genetically. Of the confirmed 49 affected individuals, we were only able to obtain detailed phenotypic information for 35 individuals due to geographical logistics and COVID restrictions. We have inserted a section within the Methods and Materials and Results sections to further clarify this to the reader.

I like that you mentioned there that these 35 are from 21 interrelated families (p.7, line 120), but maybe mention here a bit more on relatedness of individuals. Something like how many are not first-degree relatives/at least as distant as first cousins/two segregations/etc.

Unfortunately, despite the extensive genealogical records kept by the Amish, this information is not available for all of the included 35 individuals. However, we have been able to link the majority of the families (22/35 affected individuals) together into a single extensive pedigree (Figure 1F), which provides an excellent visual aid to show the degree of relatedness of these families. The remaining individuals are likely to have a similar degree of relatedness due to the genealogy of the Old Order Amish population, although this could not be ascertained explicitly.

4. “affected with SNIP1-related disorder” (p.7, line 120)…does this mean based on phenotype/clinical features? Would “suspected SNIP1-related disorder” be more accurate?

Thank you for this comment and we apologise for this lack of clarity. ‘Affected with *SNIP1*-related disorder’ refers to diagnosis made on both phenotypic findings and genotype results, and we have amended the text to ‘confirmed *SNIP1*-related disorder’ to clarify this.

5. Only a 60% probability of finding significantly differentially expressed genes in RNA-seq seems notably low. Can sample size be increased? (Authors do note that samples size is low.) Did all samples come from that one family in Figure 1F? Is there any possibility of using another individual from a different family?

We agree that the sample size is lower than we would ideally prefer. However, while we are unfortunately not able to further increase the sample size, it is important to note that these data still reach statistical significance. The six patient samples were collected from affected individuals from four separate nuclear sibships, which is now clarified in the results.

6. Regarding ClinVar (p.16, line 290): There are other VUSs as well…can you comment on predictions for these (Near NLS, FHA, etc.? Any comment on their frequency in Amish populations? (And frequency of the R111C variant?)

Thank you for this comment – there are 10 other VUS entries (6 missense) within the ClinVar database. We have not been able to obtain any information from within the Amish population which may help with interpreting potential pathogenicity of any of these other *SNIP1* variants. We have included details of the *SNIP1* (NM_024700.4:c.331C>T; p.(Arg111Cys)) variant frequency in gnomAD (allele frequency (0.00018) and no homozygotes in v2.1/v3.1) within the manuscript (line 307-8).

7. Were any other variants of interest identified in WES that were outside of the chr1 region of interest?

A major strength of the manuscript entails the uniquely powerful genetic studies stemming from the investigation of a monogenic condition at high incidence in a community setting. Due to this, we were able to exclude all other candidate gene variants such that the *SNIP1* variant remained as the sole candidate cause of disease. We have modified the text in the results to more clearly state this (‘…identified only a single potentially pathogenic sequence variant’).

8. Have you tried to identify others with variation of interest in SNIP1 through GeneMatcher?

Many thanks for this helpful comment – indeed we added *SNIP1* to GeneMatcher, unfortunately no matches have been made to date.

9. It might be nice to mention this variant is rare in gnomAD and TopMed/Bravo, with no homozygous individuals. In gnomAD, 10/11 heterozygotes are in an Amish population.

We agree with this and have detailed the gnomAD variant frequency in the results; the gnomAD frequency is very low with 10/11 heterozygous individuals listed being Amish.

**Reviewer 2:**

The authors describe in detail clinical findings in 35 individuals from the Amish community with homozygous pathogenic SNIP1 variants (c.1097A>G p.(Glu366Gly)) and define the cardinal features of the disorder. They report also on gene transcript studies in affected individuals. The paper is enjoyable, robust and well written.

The authors claim that they ‘describe extensive genetic studies and clinical findings of a complex inherited neurodevelopmental disorder in 35 individuals associated with a SNIP1 c.1097A>G p.(Glu366Gly) alteration, present at high frequency in the Amish community’. They report that ‘together these data consolidate SNIP1 gene alteration as a cause of an autosomal recessive complex neurodevelopmental disorder and provide important insight into the molecular roles of SNIP1 which likely explain the cardinal clinical outcomes in affected individuals, defining potential therapeutic targets’.

This is not an original gene discovery paper as this gene is already an OMIM morbid gene, the gene is a possible gene in DDG2P and amber on UK PanelApp following work by Puffenberger et al. (2012) which identified exactly the same pathogenic variant identified in this study (c.1097A>G p.(Glu366Gly) and carried out functional work). However, it is an original thorough careful analysis of a large number of affected individuals which gives further very important evidence that biallelic pathogenic SNIP1 variants cause a distinctive disorder (SNIP1 associated syndrome). This will be extremely important for clinicians and scientists researching in the field

We are grateful for this comment and the reviewer’s recognition of the scope and importance of our study. As indicated above we have amended the title and manuscript to more clearly reflect that our study confirms the *SNIP1* disease gene/variant association and defines the clinical features and molecular basis of the condition.

The authors claim to have defined therapeutic targets in the abstract, however this isn’t specifically referred to in the discussion (however they have carried out transcript analysis to identify perturbed pathways). This could be misread as meaning a specific molecule or receptor had been identified for targeting as opposed to large pathways which is what I think they are referring to. I think this could be reworded and referred to more clearly in the discussion

We apologise if the wording around this was unclear. We have amended the text in the abstract and closing paragraph of the discussion to more clearly state that the findings identify candidate therapeutic avenues for future research.

The authors have referenced the published literature appropriately and accurately; however, they could make it clearer that the pathogenic variant identified in this report has already been published.

As per reviewer 1, we have addressed this point by altering the title of the manuscript, and the text of the Results section to clearly state the variant is the same as that identified by Puffenberger et al. (2012).

Using ACMG criteria to classify the variant would make this paper better. This would take minimal time (less than one day)

We agree with this important point, particularly as we are aware of a number of Amish children who had previously not received a diagnosis due to the current classification of the *SNIP1* c.1097A>G p.(Glu366Gly) variant in ClinVar as a VUS. We have classified the variant according to ACMG criteria (pathogenic), detailed this in the Results and Supplemental data file and also submitted the classification to ClinVar.

It would have been helpful to give more information about the background of the Amish population and Old Order Amish as this is not given.

Thank you for this comment; we have added a little more detail to the introduction within the bounds of a scientific manuscript such as this, regarding the ancestry and migration of the Amish from Europe to the USA during the 18^th^/19^th^ centuries.

It would be helpful to clinicians to highlight the spliceosomal aspects of this gene in the abstract so they can place it in context.

We agree with the reviewer on this point and have added this to the abstract.

It isn’t totally clear how the authors identified the affected individuals. It would be helpful to state How exactly did you identify them? In clinic / examining them through the community? They refer at one point to a historical review of notes and parental survey. More clarity throughout would be helpful to the reader.

We have now included the Clinics through which the families were identified and recruited with informed consent in the Methods and Materials and Results sections.

The use of ‘alteration’ in the title and paper is not the most accurate scientific/clinical word. Likewise, the title could be improved, and the authors could consider use of ‘SNIP1-associated syndrome’ which they use later in the paper.

As described above, the title of the manuscript has now been amended, along with the use of the word ‘alteration’.

With regards to epilepsy, were there any defining aspects of the individuals’ epilepsy. Any EEG abnormalities? Photosensitivity ? febrile seizures Are there any medications that any individuals responded well to? Information about treatment is very helpful to epileptologists.

Thank you for raising these important questions. Intractable myoclonic epilepsy is common (and subclinical seizures are not uncommon) in *SNIP1*-related disorder. EEGs are always abnormal and seizures are exacerbated by several factors, including febrile illnesses, sleep deprivation, and hypoglycemic episodes, although photosensitivity has not been identified as a specific issue. There does not appear to be any specific individual medication that patients respond well to and several individuals have multi drug resistant epilepsy. We have expanded the text in the results section to accommodate these important points and included serial EEG results for 8 individuals in Supplemental data.

The authors state several individuals required more than two antiepileptic drugs to achieve ‘reasonable’ seizure control. They don’t state how they defined reasonable seizure control. Do they mean: No seizures? Family reported happy with control? Doctor happy with control? No drop attacks?

We defined reasonable seizure control as ‘a manageable frequency of seizures’. This may mean no seizures in some individuals while in others it means some seizures still occur but are manageable from the viewpoint of the family. Even with good seizure control, breakthrough seizures often still occur with intercurrent illness. We have again modified the text to clarify this.

The authors state: Individuals identified in late childhood/adolescence are often diagnosed with a mixed tone spastic quadriplegic cerebral palsy. Is this progressive? Why is this not picked up earlier?

The basis of the spastic quadriplegia in affected older individuals who had not previously come to medical attention reflects hypoxic injury stemming from uncontrolled and lengthy seizures during early childhood, and we have included text in the manuscript to reflect this. A key aspect of this manuscript is to increase recognition of the disease in the community (and globally) to enable earlier diagnosis and more effective treatment management, to reduce preventable morbidity due to late diagnosis of the condition.

The authors state: Other clinical features associated with SNIP1-related disorder include a variable spectrum of congenital cardiac defects (60% of individuals), Are the authors certain that all of these heart defects are all related to SNIP-1 and not due to other homozygous pathogenic variants in other genes? Are isolated heart defects more common in the Amish population than in the general population or not?

While it is never possible to conclusively exclude contributions to disease phenotypes from other genetic variants not identified in genomic studies, due to the high proportion of congenital cardiac defects within this group of patients (60%) and the large number of patients ascertained, it is certain that cardiovascular disease forms an important feature of *SNIP1*-related disorder.

There are a small number of typographical errors. Figure 1 looks unfinished, parts A to D are missing. These appear to be clinical photos

We apologise for our typographical errors and have now corrected these. Figure 1 includes 4 clinical photographs of affected individuals for which we have now obtained a signed PLOS Genetics consent form for publication. It is important to include these consented clinical photographs to illustrate the recognisable craniofacial features associated with the condition, to aid diagnosis of the disorder in future patients.

We hope that you agree that the amendments we have made in light of the reviewers’ comments have improved the manuscript and thank you again for considering this work for publication in *PLOS Genetics*. Please let us know if there is any further information that you require.

Yours sincerely,

Professor Andrew Crosby

*Professor of Human Genetic*

*Tel: +44 (0) 1392 408302*

[A.H.Crosby@exeter.ac.uk](mailto:A.H.Crosby@exeter.ac.uk)

Dr Emma Baple

*Consultant Clinical Geneticist*

*Snr Lecturer Genomic Medicine*

*Tel: +44 (0)1392 405725*

[E.Baple@exeter.ac.uk](mailto:E.Baple@exeter.ac.uk)

Dr Zineb Ammous

Clinical Geneticist and Medical Director

The Community Health Clinic, Topeka, IN

[zammous@indianachc.org](mailto:zammous@indianachc.org)
